# Generation of Rat-Induced Pluripotent Stem Cells from a New Model of Metabolic Syndrome

**DOI:** 10.1371/journal.pone.0104462

**Published:** 2014-08-11

**Authors:** Nana Takenaka-Ninagawa, Yuka Kawabata, Shogo Watanabe, Kohzo Nagata, Shigeko Torihashi

**Affiliations:** 1 Department of Rehabilitation Sciences, Nagoya University Graduate School of Medicine, Nagoya, Japan; 2 Department of Pathophysiological Laboratory Sciences, Nagoya University Graduate School of Medicine, Nagoya, Japan; Massachusetts General Hospital, United States of America

## Abstract

We recently characterized DahlS.Z-*Lepr^fa^/Lepr^fa^* (DS/obese) rats, derived from a cross between Dahl salt-sensitive rats and Zucker rats, as a new animal model of metabolic syndrome (MetS). Although the phenotype of DS/obese rats is similar to that of humans with MetS, the pathophysiological and metabolic characteristics in each cell type remain to be clarified. Hence, the establishment of induced pluripotent stem cells (iPSCs) derived from MetS rats is essential for investigations of MetS *in vitro*. Reports of rat iPSCs (riPSCs), however, are few because of the difficulty of comparing to other rodents such as mouse. Recently, the advantage of using mesenchymal stromal cells (MSCs) as a cell source for generating iPSCs was described. We aimed to establish riPSCs from MSCs in adipose tissues of both DS/obese rats and their lean littermates, DahlS.Z-*Lepr^+^/Lepr^+^* (DS/lean) rats using lentivirus vectors with only three factors *Oct4, Klf4*, and *Sox2* without *c-Myc*. The morphology, gene expression profiles, and protein expression of established colonies showed embryonic stem cell (ESCs)-like properties, and the differentiation potential into cells from all three germ layers both *in vitro* and *in vivo* (teratomas). Both riPSCs became adipocytes after induction of adipogenesis by insulin, T3, and dexamethasone. Real-time PCR analysis also revealed that both riPSCs and the adipose tissue from DS/obese and DS/lean rats possess similar expression patterns of adipocyte differentiation-related genes. We succeeded in generating riPSCs effectively from MSCs of both DS/obese and DS/lean rats. These riPSCs may well serve as highly effective tools for the investigation of MetS pathophysiology *in vitro*.

## Introduction

The laboratory rat (*Rattus norvegicus*) was the first mammalian species to be used for scientific research, and has been widely applied as an animal model for studies in physiology, pharmacology, toxicology, nutrition, behavior, immunology, and neoplasia [1]. The availability of many kinds of spontaneous models for diseases such as hypertension and diabetes has made the rat the preferred choice for scientific investigations. Furthermore, rats are profitable tools for transplantation studies and motor functional analysis because of their body size and ease in handling and care.

In 2006, Yamanaka *et al*. reported the generation of pluripotent stem cells from mouse somatic cells by transduction of four transcription factors (*Oct3/4*, *Sox2*, *Klf4*, and *Myc*) [2]. These cells are referred to as induced pluripotent stem cells (iPSCs). The discovery of iPSCs has contributed a great step forward in stem cell research, because iPSCs generated from patients are a great resource for novel therapeutic strategies. Moreover, iPSCs can be extremely valuable research tools, especially for rats and other species for which embryonic stem cells (ESCs) are not available or are difficult to isolate. The generation of iPSCs from disease model rats could help to clarify the pathogenesis of various disorders.

Although rat iPSCs (riPSCs) have been established recently [3]–[8], reports of their use and properties are still limited. Meanwhile, mesenchymal stromal cells (MSCs) could be used as a cell source for iPSC generation with three transcription factors, which show higher efficiency when compared with dermal fibroblasts [9], [10]. Therefore, the generation of riPSCs from rat MSCs by using three transcription factors appears to be a valid technique.

We recently established a new animal model of metabolic syndrome (MetS), the DahlS.Z-*Lepr^fa^/Lepr^fa^* (DS/obese) rat, by crossing Dahl salt-sensitive (DS) rats with Zucker rats harboring a missense mutation in the leptin receptor gene (*Lepr^fa^*). DS/obese rats develop obesity as well as hypertension, dyslipidemia, insulin resistance, and type 2 diabetes mellitus. In addition, these animals develop cardiac hypertrophy as well as renal and liver damage, which may be responsible for their premature death [11], [12], [13]. Although the phenotype of DS/obese rats is similar to that of humans with MetS, the pathophysiological and metabolic characteristics in each cell type remain to be clarified. Hence, iPSCs derived from MetS rats would promote investigations *in vitro* to resolve long-standing questions. Here, we report the establishment of riPSCs from DS/obese and DahlS.Z-*Lepr^+^/Lepr^+^* (DS/lean) rats, respectively, which can be used to investigate the cell biology of MetS *in vitro*. We generated riPSCs from MSCs collected from adult rat subcutaneous adipose tissues of DS/obese and DS/lean rats. MSCs from rats were treated with three mouse reprogramming factors (*Oct3/4, Sox2*, and *Klf4*) and enhanced green fluorescent protein (EGFP) through lentiviral vectors, according to methods described previously with some modifications [14]. The lentiviral transduction yielded ESC-like colonies from individual rat MSCs, and positive alkaline phosphatase (ALP) expression was observed in these EGFP-positive colonies, thus supporting their stem cell nature. These cells were then termed obese riPSCs (o-riPSCs) for those derived from DS/obese rats and lean riPSCs (l-riPSCs) for those derived from DS/lean rats. Like mouse ESCs, both o-riPSCs and l-riPSCs expressed stage-specific proteins and undifferentiated ESC-marker genes, and showed the capacity for differentiation.

Thus, we succeeded in generating riPSCs from MSCs of both DS/obese and DS/lean rats by using only three reprogramming factors. These riPSCs will serve as highly effective tools for studying MetS pathophysiology *in vitro*.

## Materials and Methods

### Generation of rat iPSCs

MSCs were isolated from the adipose tissues of DS/obese rats and DS/lean rats (Japan SLC; Shizuoka, Japan) according to our published protocol [15]. MSCs and feeder cells were cultured in basic-Dulbecco's modified Eagle's medium (DMEM) was used, containing DMEM (Sigma; St. Louis, MO; www.sigma-aldrich.com) with 0.1 mM non-essential amino acids (GIBCO; Carlsbad, CA; www.invitrogen.com), 1 mM sodium pyruvate (GIBCO), 1 mM 2-mercaptoethanol (Sigma), and 0.5% of an antibiotic-antimycotic (GIBCO) containing 10% fetal bovine serum (Biological Industries; Kibbuiz, Israel; www.bioind.com).

Rat iPSCs were generated from the MSCs by introducing three mouse factors (*Oct3/4*, *Sox2*, and *Klf4*) in lentiviral vectors (pCAG-HIVgp packing plasmid and CMV-VSVG-RSV-Rev Rev-expressing plasmid; kind gift from Dr. H. Miyoshi, Riken Tsukuba). The cDNAs of mouse *Oct3/4*, *Sox2*, and *Klf4* were inserted into a doxycycline (Dox)-inducible system lentiviral vector that also included *Egfp* inserted downstream from a ubiquitin-C (*Ubc*) promoter (mOKS plasmid; kind gift from Dr. T. Yamaguchi, Tokyo University).

For lentivirus production, 293FT cells (Invitrogen; Carlsbad CA; www.invitroge.com) were plated at 6×10^6^ cells per 100-mm dish in basic DMEM and incubated overnight. 293FT cells stably express the neomycin resistance gene and should be maintained in medium containing 500 µg/mL Geneticin (Invitrogen). Cells were transfected with three kinds of plasmid vectors and Lipofectamine 2000 (Invitrogen). Twenty-four hours after transfection, the supernatant of the transfect was collected and filtered through a 0.45-µm pore-size filter (Stericup & Steritop; Millipore; Billerica, MA; www.millipore.com). The filtered virus-containing supernatant was concentrated with lenti-X™ Concentrator (Clontech; Mountain View, CA; www.clontech.com). Lentiviral infection and expansion of riPSCs were conducted as previously reported with some modifications [8]. The medium for MSCs was replaced with concentrated supernatant supplemented with 4 µg/mL polybren (Nacalai Tesque; Kyoto, Japan), and incubated for 24 h. We added doxycycline (Dox) to the culture medium on the day of viral infection (day 0) and mouse leukemia inhibitory factor (LIF; Chemicon; Temecula, CA; www.millipore.com) to the medium on day 1. Based on our previous studies, we can use either rat or mouse LIF. We did not find any differences in their characteristics, including proliferation and differentiation potential, profiles of mRNA expression and cell surface markers, when we cultured riPSC in DMEM containing mouse or rat LIF (data not shown). Specifically, in this study, we used mouse LIF. Two days after lentiviral infection, transduced cells were trypsinized and split on SNL feeder cell layers (CELL BIOLABS; San Diego, CA; www.cellbiolabs.com) that were mitotically inactivated with 10 µg/mL mitomycin C (Kyowa Hakkou Kirin; Tokyo, Japan; www.kyowa-kirin.co.jp) in basic DMEM. On day 7, 1 µM mitogen-activated protein kinase kinase (MEK) inhibitor (PD0325901, Sigma) and 3 µM glycogen synthase kinase 3 (GSK3) inhibitor (CHIR99025; Sigma) were added to the medium. Eight days later (10 days after transduction), generated EGFP-positive iPSC colonies were picked up and mechanically dissociated. Dissociated iPSCs were plated into new wells with SNL feeder (24-well plates). The generated rat iPSCs ubiquitously expressed EGFP under the control of the *Ubc* promoter.

### Induction of differentiation

#### 1) Neurogenesis

Both rat iPSCs, i.e., o-riPSCs and l-riPSCs, were expanded on feeder cell layers in basic-DMEM. For the expansion of rat iPSCs, 1000 U/mL LIF was added in basic-DMEM. For neurogenesis, riPSCs were incubated for 2 days without LIF and compacted to form embryoid bodies (EBs) in hanging drops. After that, EBs were exposed to 5×10^-5^ M all-*trans* retinoic acid (RA, Sigma) in basic DMEM for 5 days and were placed in a magnetic cell separation system (MACS; Miltenyi Biotech; Auburn, CA; www.miltenyibiotec.com) by using PSA-NCAM micro beads (130-092-966 Miltenyi Biotech). The sample preparation, magnetic labeling, and magnetic separation with LS columns were conducted according to the manufacturer's instructions. Sorted neuron progenitors were attached to dishes coated with poly-d-lysine hydrobromide (P7280, Sigma)-laminin (L2020, Sigma), and then they were differentiated into motor neurons by addition of 25 ng/mL sonic hedgehog (recombinant mouse sonic hedgehog N-terminal 461-SH; R&D Systems; Minneapolis, MN; www.RnDSystems.com) and 2×10^-5^ M RA in basic-DMEM. The culture medium was changed every day.

#### 2) Adipogenesis

After expansion of each group of riPSCs, they were compacted to form EBs in hanging drops for 2 days. Over the following 3 days, they were exposed to 1×10^-6^ M RA in culture medium followed by washing for 1 day without RA. After 6 days, the EBs were plated onto gelatin-coated dishes and then incubated with 850 nM insulin (Sigma) and 20 nM 3,3,5-triiodo-l-thyronine (T3; Sigma) in basic-DMEM. MSCs expressing CD105 in developing EBs were sorted by MACS by using both an anti-CD105 antibody (R&D Systems) and an anti-rat IgG antibody conjugated with magnetic beads (Miltenyi Biotec). The sample preparation, magnetic labeling, and magnetic separation with mini-MS columns were conducted according to the manufacturer's instructions. After MACS separation, CD105+ MSCs were transferred to gelatin-coated dishes. For further induction to adipocytes, they were maintained in 850 nM insulin, 20 nM T3, and 1 µM dexamethasone (Dex; D8779, Sigma) in basic DMEM. In addition, 16 µM recombinant rat leptin (400-21; PeproTech, Rocky Hill, NJ) was supplemented for some MSCs from both groups of rat iPSCs, i.e., o-riPSCs and l-riPSCs. The culture medium was changed every day.

### Histological and immunofluorescent staining

#### 1) Alkaline phosphatase

Cells were fixed with 4% paraformaldehyde for 15–30 minutes. Histochemical ALP staining was processed using Vector Red Alkaline Phosphatase Substrate Kit I (Vector Laboratories,Burlingame, CA).

#### 2) BODIPY staining

For lipid staining, 2 µM BODIPY 558/568 C_12_ (D-3835, Molecular probes, Leiden, Netherlands; www.probes.com) were added in the induction medium of adipose cells for 24–48 hours, and fixed with 4% paraformaldehyde after rinsed by PBS.

#### 3) Immunofluorescent staining

Cells or tissues were fixed with 4% paraformaldehyde. Tissues were frozen and cryosections were used. Primary antibodies shown in Table 1 were applied on cells or cryosections followed by appropriate secondary antibodies conjugated with Alexa Fluoro 488 and/or 594 (Molecular Probes), also listed in Table 1, and mounted with Permafluor™ Aqueous mounting medium (TA-030-FM; Thermo; Fremont, CA) just after being counter-stained with DAPI (71-03-01 KPL; Gaithersburg, MD). Samples were imaged using a microscope BZ-9000 (KEYENCE; Osaka, Japan; www.keyence.co.jp), and were recorded and analyzed with a BZ-II analysis application (KEYENCE).

**Table 1:** Antibodies for immunostaining.

Antigen	Antibody	Species	Dilution
Cdx2	CDX2-88 BioGenex www.scbt.com	Mouse monoclonal	X 1000
MHC	A4.1025 Upstate; www.upstate.com	Mouse monoclonal	X 200
Tuj-1	TuJ-1 R&D systems;	Mouse monoclonal	X 100
	www.RnDSystems.com		
type 2 collagen	LSL CO., LTD., www.cosmobio.co.jp	Rabbit polyclonal	X 200
EGFP	Novus Biologicals Inc.;	Rabbit polyclonal	X 100
	www.NovusBIo.com		
SSEA-1	Santacruz; www.scbt.com	Mouse monoclonal	X 100
Nanog	Santacruz; www.scbt.com	Rabbit polyclonal	X 100

The secondary antibody was Alexa Fluor 594 goat anti-rat IgG (1∶400; Molecular Probes, Leiden, Netherlands; www.probes.com), Alexa Fluor 594 goat anti-mouse IgG (1∶400; Molecular Probes), Alexa Fluor 488 goat anti-mouse IgG (1∶400; Molecular Probes), Alexa Fluor 594 goat anti-rabbit IgG (1∶400; Molecular Probes), or Alexa Fluor 488 anti-rabbit IgG (1∶400; Molecular Probes).

#### 4) Polymerase chain reaction (PCR) analysis

Total mRNAs from cells were extracted with the RNeasy micro kit (Qiagen; Valencia, CA; www.quiagen.com) according to the manufacturer instructions. RNAs were immediately reverse-transcribed to cDNA using SuperScript™ II (Qiagen). The primer pairs, annealing temperature, and cycling conditions used for PCR are shown in Table 2.

**Table 2:** Primers for RT-PCR.

Gene	Primer Sequences	Annealing Temp. °C	Cycles	Product Size. bp
*Transgene T2A*	Fw: GGAAGTCTGCTAACATGCGGTG	54	36	200
	Rv: GGCCATACCATGAAGGCGTTCAT			
Rat *Oct3/4*	Fw: CGAGGCCTTTCCCTCTGTTCCT	62	39	119
	Rv: TCTCTTTGTCTACCTCCCTTCCTTGC			
Rat *Klf4*	Fw: CAGACCTGGAAAGTGGTGG	58	39	283
	Rv: ACCTGTGTTGCCCGCAGCC			
Rat *Sox2*	Fw: GGCCATTAACGGCACACTGCC	62	39	120
	Rv: TTACTCTCCTCTTTTGCACCCCTCC			
Rat *Eras*	Fw: CGAGCGGTGTGGGTAAAAGTG	50	36	501
	Rv: GGTGTCGGGTCTTCTTGCTTG			
*Egfp*	Fw: ATGGTGAGCAAGGGCGAG	58	36	249
	Rv: AGTCGTGCTGCTTCATGTGG			
*β-actin*	Fw: CATGGCATTGTGATGGACT	53	36	427
	Rv: ACGGATGTCAACGTCACACT			
*Sox17*	Fw: GGCACGGAACCCAACCAGC	72	36	210
	Rv: CAGTCGTGTCCCTGGTAGGGAAGAC			
*Ncam*	Fw: TGCTCAAGTCCCTAGACTGGAACG	72	39	413
	Rv: CTTCTCGGGCTCTGTCAGTGGTGTGG			
*SM22-a*	Fw: GCTGAAGAATGGCGTGATTCTGAG	62	36	256
	Rv: CCTTCAAAGAGGTCAACAGTCTGG			
*Leptin-R*	Fw: AACAGCAAAATGATGCAGGG	62	36	322
	Rv: GATGCTCAAATGTTTCAGGC			

Total mRNAs from adipose tissues were extracted with the RNeasy mini kit (Qiagen). Portions of the RNA (2 µg) were subjected to reverse transcription (RT) with the use of a PrimerScript RT Reagent Kit (Takara; Shiga, Japan; www.takara-bio.co.jp). Quantitative RT-PCR analysis was performed with the use of SBYR Mix Ex Taq II (Takara), a Thermal Cycler Dice Real Time System II (Takara), and the specific primers for cDNAs encoding peroxisome proliferator–activated receptor γ (PPARγ) and adiponectin, shown in Table 3. Reagents for detection of glyceraldehyde-3-phosphate dehydrogenase (*Gadph*) mRNA (Applied Biosystems; Foster City, CA, USA) were used to quantify rat *Gadph* mRNA as an internal standard. Data on *Ppparγ* and adiponectin mRNAs were normalized by the amount of *Gadph* mRNA and expressed relative to the mean value for DS/lean rats. Annealing temperature was 60°C and the cycling condition was 42 cycles.

**Table 3:** Primers for real time PCR.

Gene	Primer Sequences	Product Size. bp
*Pparγ*	Fw: TGACCTGAAGCTCCAAGAATACC	97
	Rv: ATGTGGCCTGTTGTAGAGTTGG	
*Adiponectin*	Fw: GCCCTACGCTGAATGCTGAG	69
	Rv: GAACCCCTGGCAGGAAAGG	

Annealing temperature was 60°C and cycling condition was 42 cycles.

### Teratoma formation

For the transplantation of rat iPSCs, 8-week-old severe combined immunodeficient mice (SCID) were purchased from Japan Charles River (Yokohama, Japan; www.crj.co.jp) and used following the guidelines for the Animal Care and Use of the Nagoya University Graduate School of Medicine (permit number: 023-020). The committee specifically approved these animal studies. Mice were anesthetized with diethyl ether and injection of **sodium pentobarbital** (40 mg/kg body weight) (**Somnopentil**; Schering-Plough **Animal Health**, USA; www.merck-animal-health.com).

Four independent iPSC-like colonies that were derived from a single mesenchymal cell from each of the obese and lean rats were subjected to this assay.

Either o-riPSCs or l-riPSCs (3×10^6^ cells) were injected into the tibialis anterior muscles of SCID mice. Five weeks after transplantation, all mice were humanely killed and the epidermis was cut and opened to expose the anterior tibialis muscles. The transplanted areas were then observed under a dissection microscope. Transplanted muscles were fixed with 4% paraformaldehyde and then frozen. Muscle cryosections (10-µm-thick) were obtained using a cryostat. Some sections were stained with hematoxylin and eosin (H-E) and others were processed for fluorescent immunostaining.

## Results

### Expression of pluripotency markers in rat iPSCs

**Figure 1:**
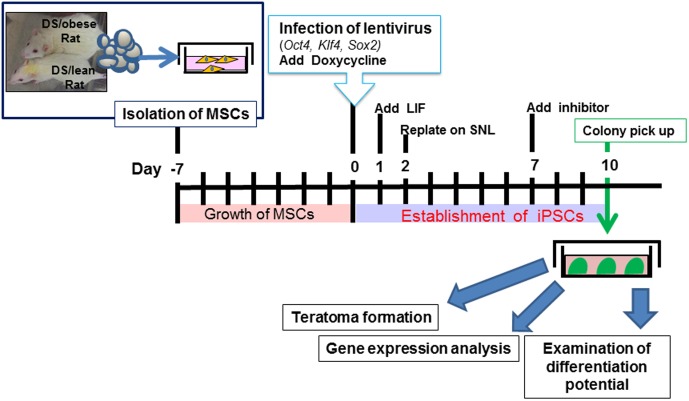
Time schedule of iPSC generation. The experiment was composed of three main processes. The first process was isolation of MSCs from the adipose tissue of either DS/obese or DS/lean rats. The second process was infection of lentivirus. The final process was the selection of iPSC colonies. The collected MSCs were cultured for about 7days. When the MSCs increased in number, we infected them with a lentiviral vector carrying three mouse reprogramming factors, and termed this time point as day 0. The MSCs were then cultured in medium for 2 days, and the cells were replated on SNL feeder cell layers. Finally, EGFP-positive riPSC colonies were picked up at day 10 and some of clones were selected for further analysis.

To generate riPSCs, we initially infected MSCs isolated from the adipose tissues of DS/obese rats and DS/lean rats, respectively, with a lentiviral vector carrying three mouse reprogramming factors (*Oct3/4*, *Sox2*, and *Klf4*). They were controlled by a tetracycline-responsive regulatory element and a *Ubc* promoter-driven reverse tetracycline transactivator containing EGFP. We added Dox to the culture medium from the day of infection. Both infected and non-infected MSCs were seeded onto mitomycin C-treated SNL feeder cell layers (Fig. 1).

**Figure 2:**
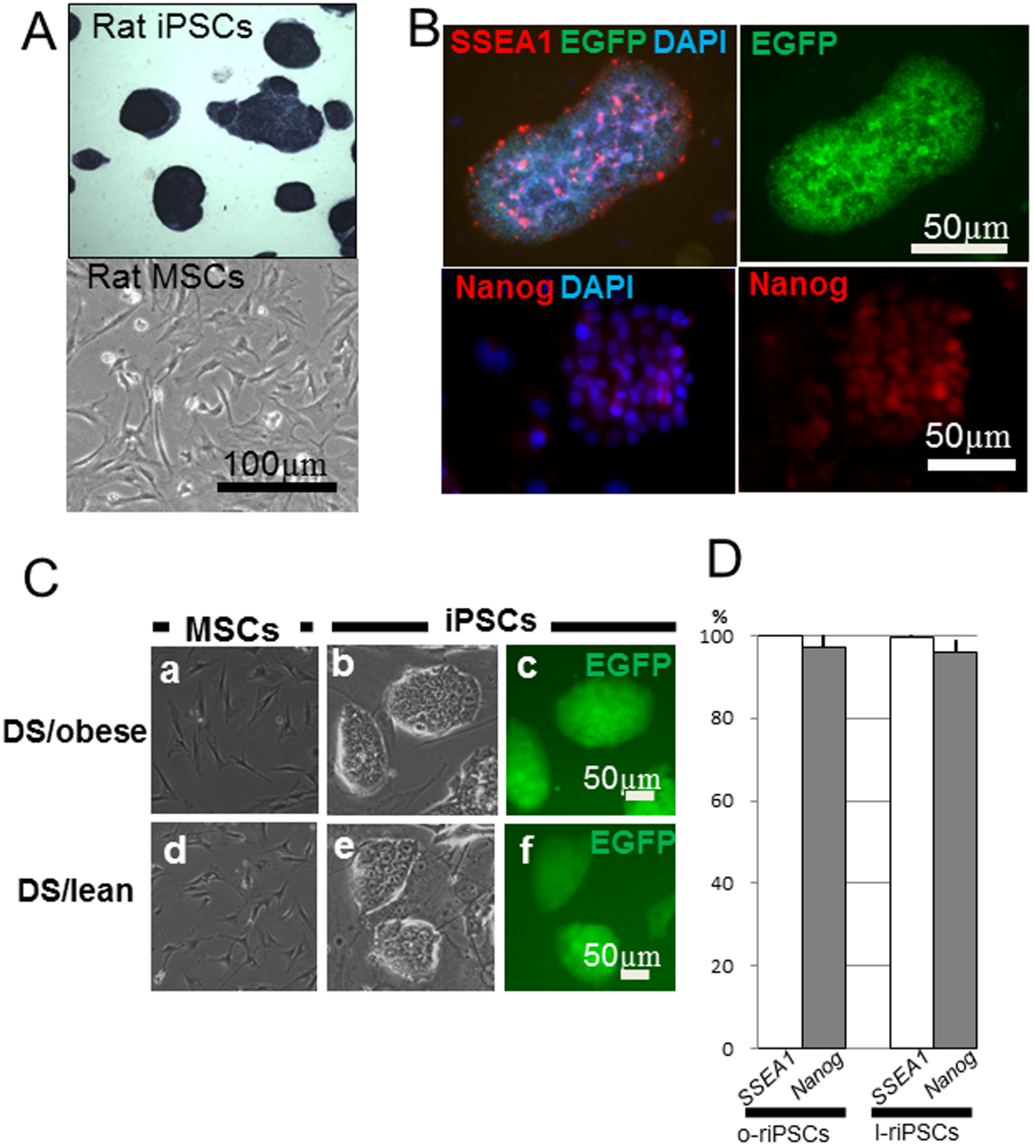
Formation of iPSC colonies. A) The ESC-like colonies were generated from rat MSCs by lentiviral transfection. These colonies were positive for alkaline phosphatase (ALP) staining (upper panel), whereas the non-transfected rat MSCs could not form any colonies (lower panel). B) Generation of riPSCs was confirmed by the expression of SSEA-1 (red) and EGFP (green in the upper panels). These riPSC colonies also expressed Nanog (red in the lower panels). C) Both MSCs derived from DS/obese (a) and DS/lean (d) rats showed a bipolar shape, while colonies of both o-riPSCs (b, c) and l-iPSCs (e, f) formed clusters expressing EGFP, showing a similar appearance to ESCs. D) The ratio of clones expressing pluripotency markers (SSEA-1 or Nanog) was demonstrated by counting the number of total pluripotency marker-positive colonies per the total number of EGFP-positive colonies. Almost all established EGFP-positive clones expressed both of pluripotency markers, respectively.

Morphologically ESC-like colonies appeared 10 days after transfection. They expressed EGFP, stage-specific embryonic antigen (SSEA)-1, and Nanog (Fig. 2B). EGFP-positive colonies expressed ALP (Fig. 2A). Colonies from DS/obese rats and DS/lean rats showed similar appearance (Fig. 2C). Furthermore, more than 95% of established EGFP-positive colonies showed distinct key features of rESCs, such as expression of pluripotency markers (Fig. 2D). However, MSCs that were not transfected with the reprogramming factors could not generate any colonies expressing EGFP, even though they were cultured under the same conditions for 10 days. These results indicate that the cells generated from DS/obese and DS/lean MSCs by lentiviral transfection were iPSCs, i.e., riPSCs.

Eight days later (10 days after transduction), several EGFP-positive riPSC clones from both DS/obese and DS/lean MSCs were picked up and mechanically dissociated. Dissociated iPSCs were plated into new wells with SNL feeder cells (24-well plates) and some of the clones were selected for further analysis (Fig. 1).

**Figure 3:**
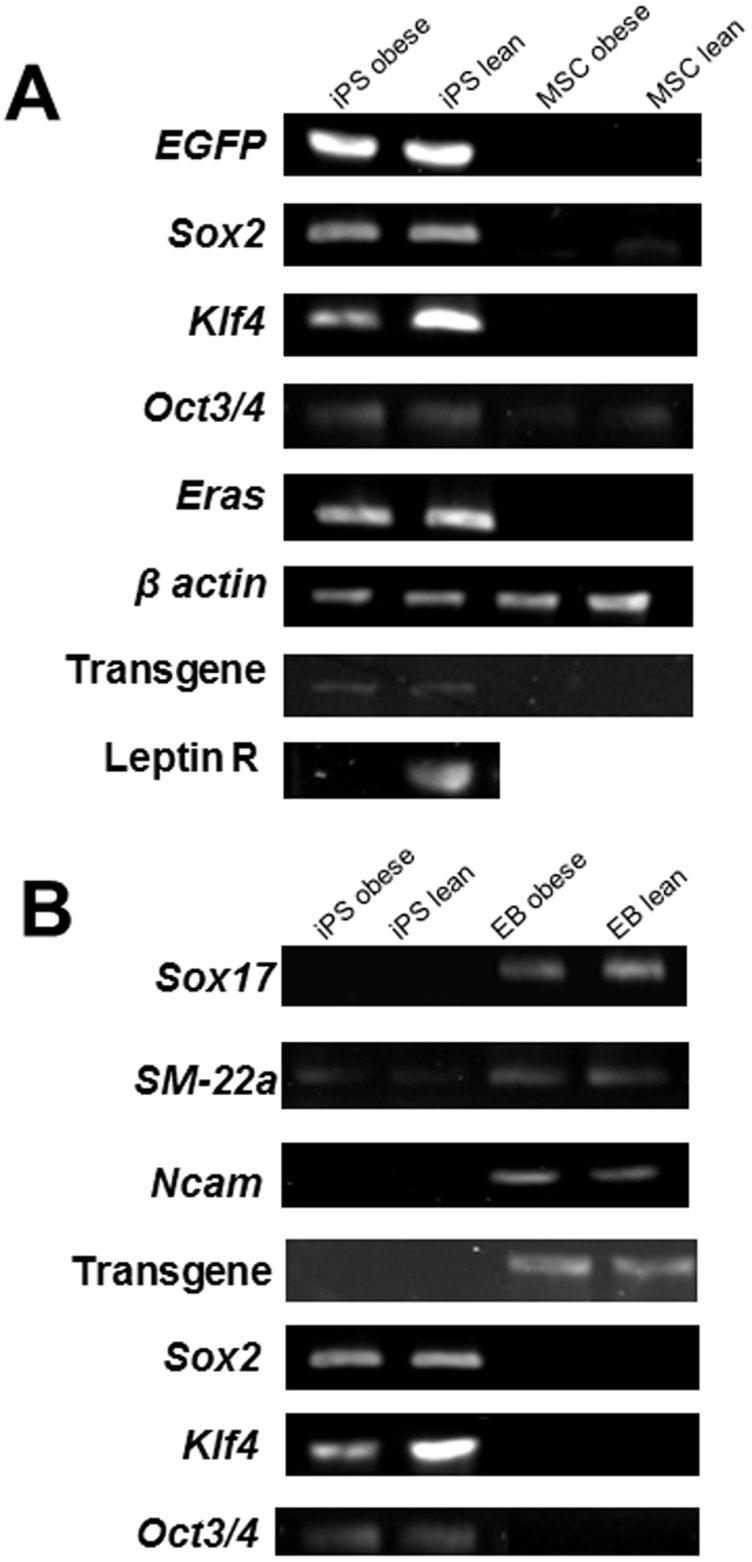
mRNA expressions. The expression of mRNAs was analyzed by RT-PCR. A) Introduced four genes (*EGFP, Sox2, Klf4 and Oct3/4*) were expressed in both o-riPSCs and l-riPSCs, but not in both MSCs. Transgenes (mouse mRNA) were detected only in undifferentiated riPSCs cultured in medium containing Dox. Leptin receptor was not detected in the o-riPSCs, however, it was expressed in thel-riPSCs. B) After differentiation in embryoid bodies (EB), *Sox17, SM-22a*, and *Ncam* were clearly demonstrated in both o-riPSCs and l-riPSCs. Introduced genes (*Sox2, Klf4* and *Oct3/4*) and Transgenes (mouse mRNA) were not detected in differentiated embryoid bodies (EB) of both o-riPSCs and l-riPSCs.

RT-PCR showed that the riPSCs expressed many undifferentiated ESC-marker genes, including *Sox2*, *Oct3/4*, *Klf4*, and *Eras*. Although *Sox2*, *Klf4*, and *Eras* were not detected in non-induced MSCs, low-level expression of *Oct3/4* was detected in non-induced MSCs. The primers used to amplify the sequence between T2A and *Sox2* were used to detect transgene expression. Transgenes were detected only in conditions with Dox and were not detected in non-induced MSCs or differentiated riPSCs, indicating that expression via the lentiviral vector was controlled by a tetracycline-responsive element. Leptin receptor was not detected in the riPSCs from DS/obese rats (o-riPSCs), although it was expressed in the riPSCs from DS/lean rats (l-riPSCs) (Fig. 3A).

In order to determine the pluripotency of both o-riPSCs and l-riPSCs *in vitro*, we allowed them to differentiate for 2 weeks and analyzed the presence of differentiation markers. RT-PCR confirmed that the riPSCs could differentiate into all three germ layers in EBs, as evidenced by the expression of SRY box containing gene 17 (*Sox17*, endoderm), *SM22-a* (mesoderm), and *Ncam* (ectoderm) (Fig. 3B). In contrast, exogenous mouse gene expression and undifferentiated ESC-marker gene expression levels were markedly decreased in differentiated riPSCs (Fig. 3B). These data indicate that the riPSCs can differentiate into three germ layers *in vitro*.

### Differentiation potential of rat iPSCs

**Figure 4:**
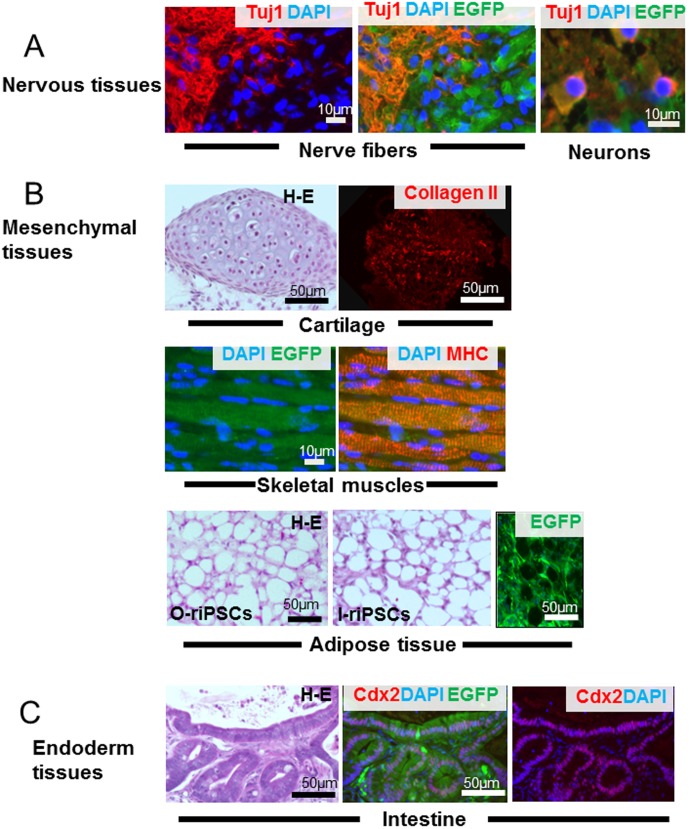
Teratoma formation. Teratomas that grew in the anterior tibialis muscles were morphologically analyzed. A) Nerve fibers and neurons expressing TUJ-1 (red) immunoreactivity were identified clearly (ectoderm tissue). B) As mesodermal tissues, cartilage and skeletal muscles that expressed EGFP (green) were detected. The cartilage showed type II collagen (red) immunoreactivity and the skeletal muscles formed myosin heavy chain-positive bundles (red). Adipose tissues in H-E staining from o-riPSCs and l-riPSCs demonstrated similar morphological features, and their serial frozen sections showed EGFP expression, indicating that they were indeed riPSCs. C) Intestinal epithelium (endoderm tissue) expressing CDX-2 (red) developed in the teratoma.

Rat iPSCs were injected into the anterior tibialis muscles of SCID mice. Both groups of rat iPSCs, i.e., o-riPSCs and l-riPSCs, generated teratomas 5 weeks after transplantation. Histological examination showed that the tumors contained various tissues originating from the three germ layers (Fig. 4).

In the teratoma, nerve fibers and neurons expressing TUJ-1 immunoreactivity were clearly identified (ectoderm tissue) (Fig. 4A). For mesodermal tissues, cartilage and skeletal muscles expressing EGFP were detected. Cartilage showed type II collagen immunoreactivity, and the skeletal muscles formed myosin heavy chain-positive bundles. Since skeletal muscle cells of the host SCID mouse did not express EGFP, muscle cells from rat iPSCs were easily identified in the anterior tibialis muscle (Fig. 4B). In the adipose tissues expressing EGFP, adipocytes showed almost the same morphological features between o-riPSCs and l-riPSCs (Fig. 4B). The average diameter of the long axis of 100 randomly selected H-E-stained fat droplets from o-riPSCs was 23.3±9.09 µm and was 24.5±13.9 µm from l-riPSCs, even though shrinkage occurred in the H-E-stained samples. Comparisons between groups were assessed by the Student's *t*-test in Excel and the difference was not statistically significant. Smooth muscle fibers were also observed (data not shown). Intestinal epithelium (endoderm tissue) expressing CDX-2 developed in the teratoma (Fig. 4C); some of these tissues also showed HNF3β/FOXA2 immunoreactivity (data not shown). These results indicate that the generated rat iPSCs had been reprogrammed into a pluripotent state like ESCs, and showed the potential for differentiation into three germ layers.

**Figure 5:**
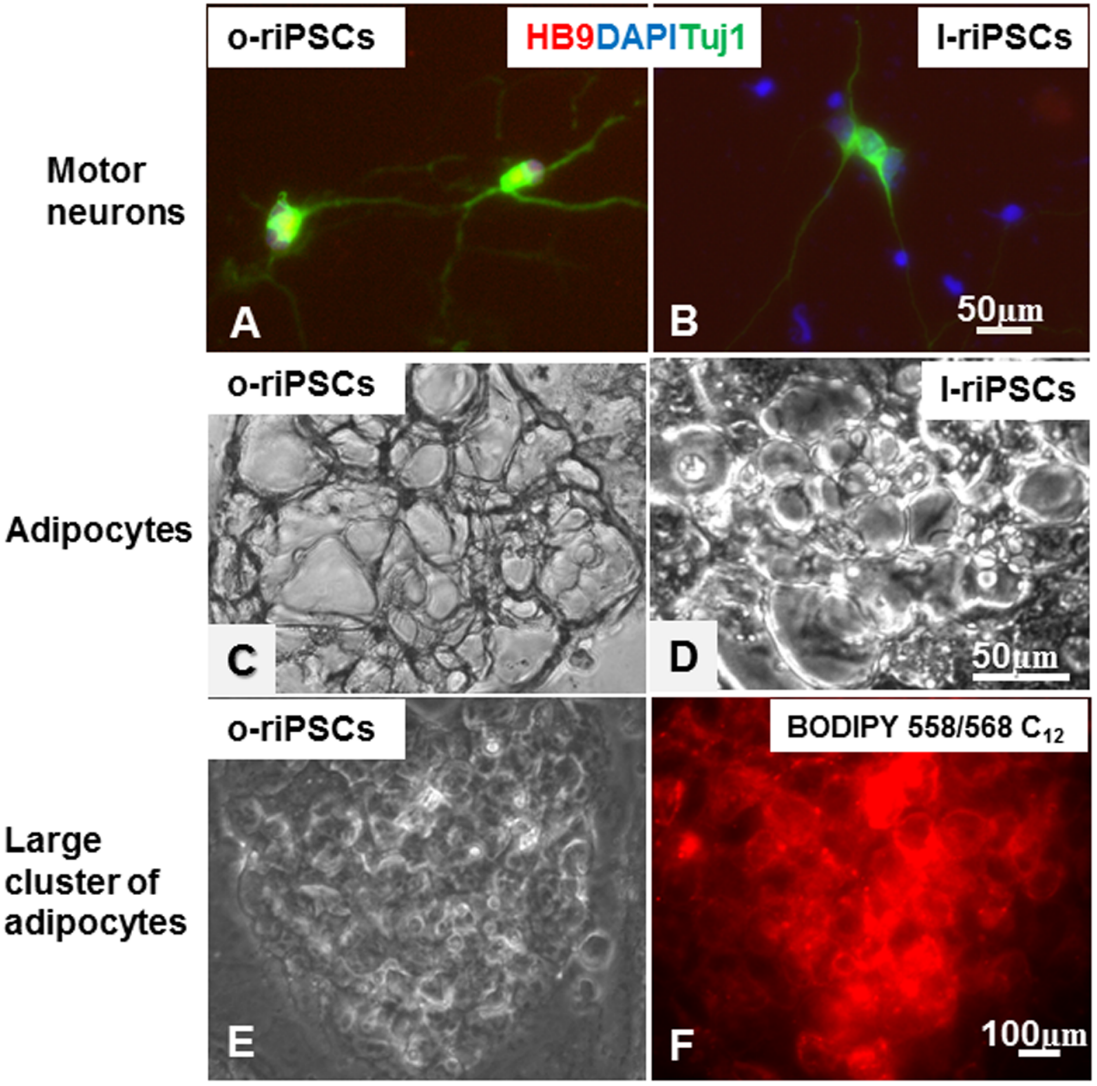
Potential for differentiation into motor neurons and adipocytes. A, B) Both o-riPSCs and l-riPSCs differentiated into motor neurons expressing HB9 (red) and TUJ-1 (green). They had long axon-like processes. C, D) Both groups of riPSCs showed the potential for adipogenesis. Their appearances were similar in size and cell number. E) Growing adipocytes formed large clusters similar to fat tissues. F) The mature fat cells were detected by involved BODIPY 558/568 C_12_ (red) that was added to the culture medium for 24–48 h.

**Figure 6:**
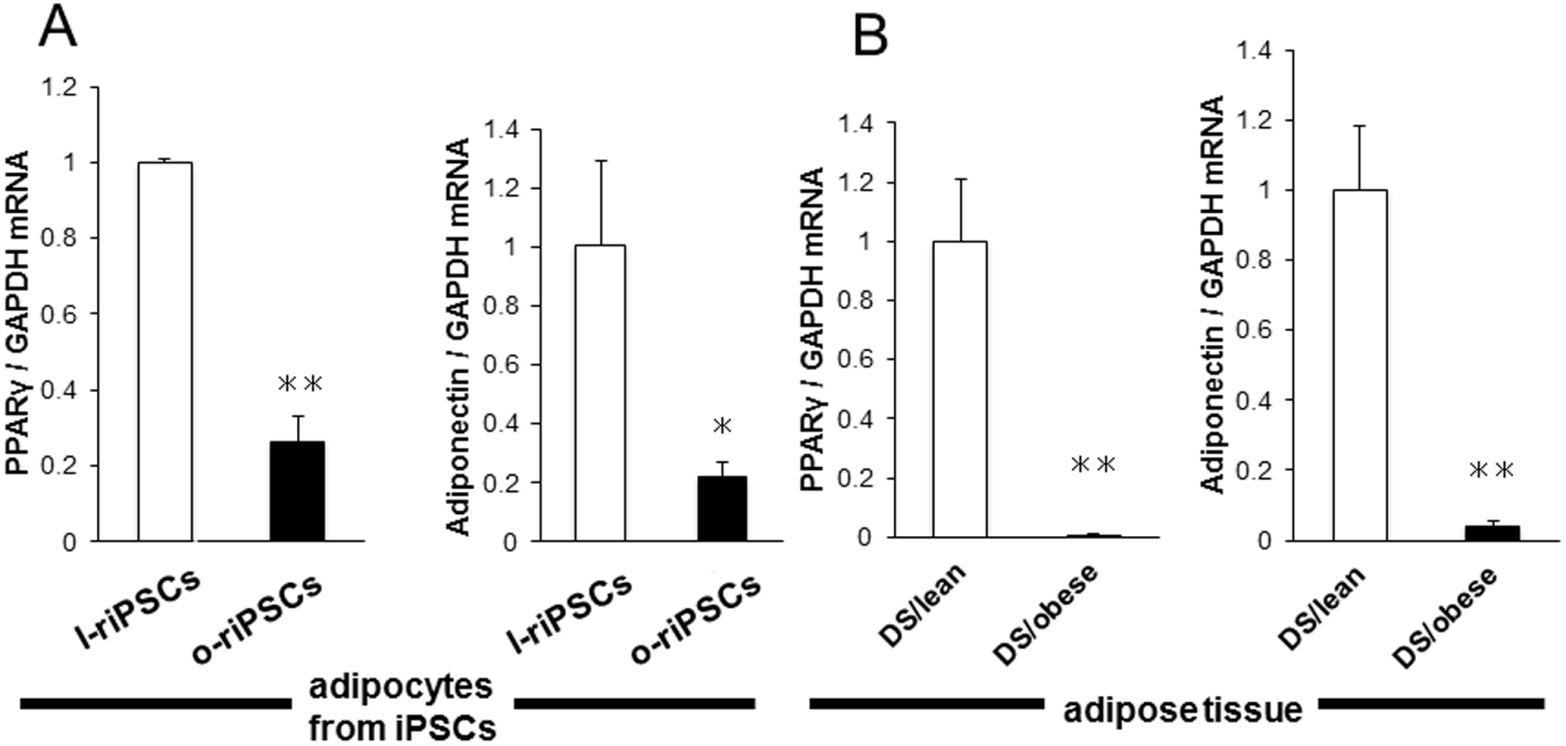
Expression of adipocyte differentiation-related genes in adipocytes from riPSCs and from DS/obese and DS/lean rats. A) Quantitative RT-PCR analysis of peroxisome proliferator-activated receptor γ (*Pparγ*) and adiponectin mRNAs in adipocytes differentiated from l-riPSCs and o-riPSCs. B) *Pparγ* and adiponectin mRNA from DS/obese and DS/lean rats. Data are means ± SEM of values from two independent experiments for adipocytes differentiated from riPSCs (*n* = 4 each; **A**) and for animals (n = 5 and 4 for DS/lean rats and DS/obese rats, respectively; **B**). *P<0.05 versus DS/lean, **P<0.01 versus DS/lean.

The potential for differentiation into neurons and adipocytes was also evaluated *in vitro*. Both o-riPSCs and l-riPSCs differentiated into motor neurons expressing HB9 after induction of neurogenesis (Fig. 5AB). In addition, both groups of riPSCs differentiated into adipocytes after induction of adipogenesis by insulin, T3, and Dex. Rat iPSCs formed adipocytes after induction for 5 days (Fig. 5CD). Adipocytes including BODIPY 558/568 C_12_ were illuminated under fluorescence (Fig. 5EF). Real-time PCR analysis revealed that the mRNA levels of *Pparγ* and adiponectin (adipocyte differentiation markers) were decreased in adipocytes differentiated *in vitro* from o-riPSCs compared with those from l-riPSCs (Fig. 6A). We also confirmed that the expression of *Pparγ* and adiponectin mRNAs in the adipose tissues was decreased in DS/obese rats compared with DS/lean rats (Fig. 6B). These results indicate that both adipocytes from riPSCs *in vitro* and the adipose tissue from DS/obese and DS/lean rats *in vivo* possess similar expression patterns of adipocyte differentiation-related genes.

## Discussion

We here present evidence that rat MSCs could be reprogrammed efficiently by transduction of three transcription factors, mouse *Oct3/4*, *Sox2*, and *Klf4*, using a lentivirus. These reprogrammed rat cells are very similar to mouse ESCs. They can differentiate into cells originating from all three germ lineages *in vitro* and form teratomas *in vivo*. Reprogrammed cells were maintained for more than 15 passages, and still exhibited similar differentiation potential afterward, as has been observed for ESCs both *in vitro* and *in vivo*. We then concluded that our reprogrammed rat cells were novel riPSCs. Like mouse ESCs, long-term maintenance of riPSCs required the presence of LIF in the culture medium. Under feeder-free conditions, however, LIF could neither inhibit the differentiation nor maintain the expression of SSEA-1 and Nanog protein (data not shown).

Reports of the generation of rat iPSCs are scarce, and the reprogramming efficiency of rat somatic cells reported previously has been low. In this study, however, the efficiency of lentiviral transfection into MSCs derived from the adipose tissue was high. Furthermore, almost all established EGFP-positive clones showed distinct key features of mouse ESCs, such as expression of pluripotent and self-renewal markers, and the capacity for differentiation into derivatives of all three germ layers.

Our protocol implicates two main elements in the generation of riPSCs. The first element is the importance of appropriate virus selection. Rat somatic cells were not reprogrammed by a retrovirus, but were rather successfully established by lentiviral transduction [4]. The protocol we used was the same as reported previously for the lentiviral transduction system [14]. The second key element for riPSC generation is appropriate selection of a somatic cell type for induction of pluripotency. The somatic cell type showed a significant influence on the efficiency of iPSC generation and the level of reprogramming. In general, iPSC lines derived from different somatic cell sources vary greatly in their ability to differentiate into a variety of cell types [16]–[21]. The state of differentiation of a cell is considered to correlate with a specific epigenetic profile [22]. Indeed, adult stem cells, which are multipotent, such as hematopoietic, neural, and mesenchymal stem cells (especially those derived from adipose tissues), have been described as more easy to reprogram to PSCs than terminally differentiated cell types [10], [23]. For the expression of pluripotent genes, *Oct4* is the key factor. In the present study, non-induced MSCs derived from both types of rats showed expression of *Oct4*, although the expression level was low. This might be one of the reasons for the success of iPSC generation from rat cells.

We established the riPSCs from a MetS rat model (DS/obese) and the control, homozygous, lean littermates (DS/lean). Morphological and quantitative RT-PCR data clearly showed that both o-riPSCs and l-riPSCs differentiated into adipocytes after induction of adipogenesis. However, microscopic observations indicated that the ability of both groups of riPSCs to store fat was not sufficiently high, suggesting that the induced adipocytes were relatively immature. This idea is supported by the fact that the expression level of *Pparγ* and adiponectin genes were substantially lower in adipocytes from o-riPSCs and l-riPSCs than in the adipose tissue from DS/obese and DS/lean rats. Adipose tissues in teratomas from o-riPSCs and l-riPSCs showed similar morphological features, and adipogenesis using both riPSCs *in vitro* resulted in no differences between the two groups. These data suggest that physiological and/or metabolic differences in surrounding adipose tissues between DS/obese and DS/lean rats *in vivo* had a greater effect on the adipocytes than on expression of their own genes. Further studies are required to test this hypothesis.

riPSCs derived from DS/obese rats (o-riPSCs) can be used to study pathophysiological and metabolic characteristics *in vitro*. Their potential applications would be to evaluate diseases and perform *in vitro* screening for prospective therapeutics. For *in vitro* disease modeling and screening, a large number of cells are necessary to generate and reproduce experimental data. However, almost none of the types of somatic cells derived from adult patients were able to proliferate indefinitely and maintain a similar level of quality throughout their limited lifespan. Therefore, iPSCs from patients could help promote the establishment of disease models, and lead to a better understanding of disease development. Furthermore, drug-screening platforms will help resolve incongruent drug treatment efficacies, and will hopefully lead to the future development of treatments and cures for diseases.

Furthermore, riPSCs derived from DS/lean rats (l-riPSCs) have a wide range of potential applications. For a transplantation study like that of mouse iPSCs, the recovery of injured or disordered rats could be evaluated after transplantation of l-riPSCs showing normal phenotype. Such analyses, especially with respect to behavior or locomotion, will be easier in rats than in mice due to their larger body size. The generation of riPSCs from the MSCs we used will also help in the development of other human disease model rats for the establishment of original riPSCs.

In summary, we succeeded in establishing MetS model riPSCs using MSCs. The novel riPSCs showed high potential for differentiation into all three germ layers. MetS model riPSCs offer a promising new and potentially powerful tool for investigations and progress in understanding MetS at the cellular level.
